# Concurrence of IgG4-related disease and Kimura disease with pulmonary embolism and lung cancer: a case report

**DOI:** 10.1186/s12890-022-02094-9

**Published:** 2022-08-09

**Authors:** Ye Lu, Junxiu Liu, Hengyi Yan, Wei Feng, Li Zhao, Yu Chen

**Affiliations:** 1 Department of Pulmonary and Critical Care Medicine, Shengjing Hospital of China Medical University, Shenyang, China; 2 Department of Intensive Care Unit, The Fourth People’s Hospital of Shenyang, Shenyang, China

**Keywords:** IgG4-related disease, Kimura disease, Asthma, Pulmonary embolism, Central diabetes insipidus, Lung squamous carcinoma

## Abstract

**Background:**

Immunoglobulin G4 (IgG4)-related disease (IgG4-RD) is a systemic disease that involves the infiltration of IgG4-positive plasma cells in multiple organs. Kimura disease (KD) presents as subcutaneous masses on the head and neck, frequently accompanied by eosinophilia and high immunoglobulin E (IgE) levels. Here, we report a rare case of concurrence of IgG4-RD and KD with manifestations of asthma, pulmonary embolism, and central diabetes insipidus accompanied by lung carcinoma.

**Case presentation:**

A 65-year-old Chinese male with an eight-year history of KD was admitted to our hospital with complaints of dyspnea and expectoration for one month. Laboratory examination showed a considerable elevation in the serum eosinophil count and total IgE and IgG4 levels. Chest enhanced computed tomography showed filling defects in the right pulmonary artery and a nodule in the left inferior lobe. Pancreatic enhanced magnetic resonance imaging (MRI) and magnetic resonance cholangiopancreatography showed a swollen pancreatic tail and local stricture of the pancreatic duct section of the common bile duct. Enhanced MRI of the pituitary gland showed thickening of the pituitary stalk. Additionally, immunohistochemistry of the specimens collected eight years prior revealed IgG4-positive cells. Following the diagnosis of IgG4-RD with KD, glucocorticoids with immunosuppressants were initiated; there was a prompt improvement in the patient’s condition. One-year post-discharge, the patient underwent wedge-shaped resection of the lung due to enlargement of the pulmonary nodule, and the pathology revealed lung squamous carcinoma.

**Conclusions:**

This case presents a rare clinical condition in which the concurrence of IgG4-RD and KD causes various rare manifestations including asthma, pulmonary embolism, central diabetes insipidus, and complicated lung carcinoma. This highlights the importance of monitoring for malignancies in IgG4-RD patients during follow-up.

## Background

Immunoglobulin G4 (IgG4)-related disease (IgG4-RD) is a systemic inflammatory disease that usually involves multiple systems; it is characterized by elevated serum IgG4 levels, infiltration of IgG4 positive plasma cells, and tissue fibrosis and sclerosis. It often affects multiple organs such as the pancreas, biliary tree, aorta, retroperitoneum, lung, salivary and lacrimal glands, thyroid, kidney, meninges, pituitary gland, and other organs. The pathological features are lymphoplasmacytic infiltration of IgG4-positive plasma cells with storiform fibrosis and obliterative phlebitis [1]. KD is a rare, chronic inflammatory disease of unknown etiology, and its prominent clinical features include subcutaneous masses on the head and neck, accompanied by elevated serum IgE levels, peripheral blood eosinophilia, and histopathological characteristics, including preserved nodal architecture, florid germinal center hyperplasia, eosinophilic infiltration, postcapillary venule proliferation, and eosinophilic abscesses [2]. A link between IgG4-RD and KD was suggested several times [3–9]; however, coexistence of IgG4-RD and KD in a patient with features of multiple system involvement and pulmonary embolism is exceptionally rare. In addition, in recent years, there is an increase in the incidence of malignancies in patients with IgG4-RD [10]. However, the relationship between IgG4-RD and malignancies is still unclear. We herein report an interesting case of concurrence of IgG4-RD and KD. We analyzed the reasons underlying the complex and diverse clinical manifestations of the patient to improve the understanding of the diseases.

## Case presentation

**Fig. 1:**
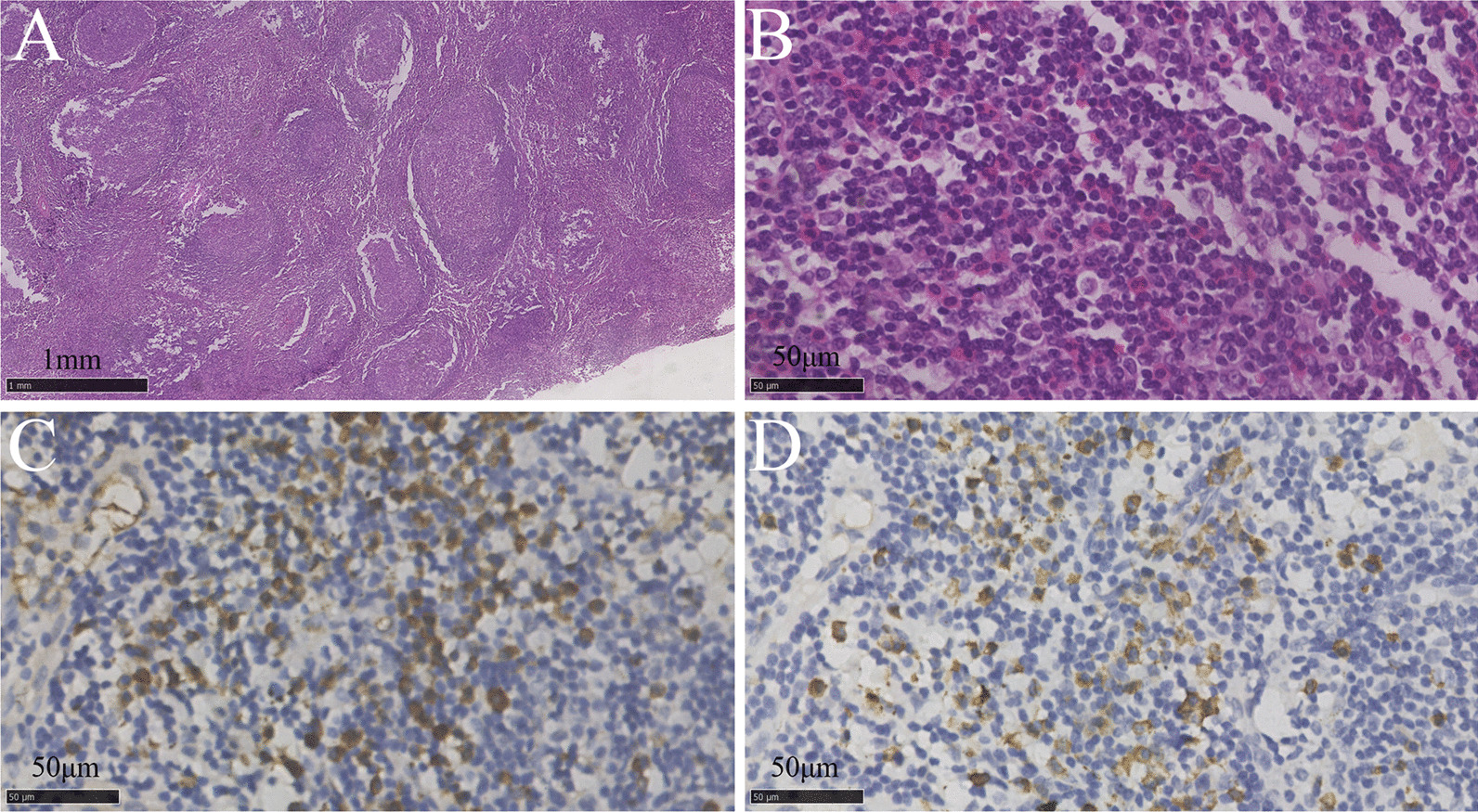
Pathologic histology of the resected right submandibular mass. **A, B** Hematoxylin and eosin (HE) staining showed hyperplasia follicles with expansion of germinal centers (a, magnification 20 ×) and eosinophilic infiltration in interfollicular region (b, magnification 400 ×). **C** Immunohistochemistry for IgG showed numerous IgG-positive cells (magnification 400 ×). **D** Immunohistochemistry for IgG4 showed an increased number of IgG4-positive cells (magnification 400 ×). The sections were observed with Nano Zoomer Digital Pathology Image. Pictures were taken by Software: NDP.view2 Plus at a resolution of 300dpi. No downstream processing was utilized

A 65-year-old Chinese male with an eight-year history of KD was admitted to our hospital on October 16, 2018, with chief complaints of cough and dyspnea over the previous month. The patient was first admitted to the department of stomatology of our hospital due to an enlarged painless right submaxillary mass in August 2010. The histopathological examination of the submaxillary mass showed preserved nodal architecture, florid germinal center hyperplasia, and eosinophilic infiltration (Fig. 1A, B). The pathological findings were consistent with KD. The patient underwent surgery without any other treatment. Five years prior to admission, his right submaxillary mass reappeared and enlarged progressively. However, he did not attach importance to the symptom and did not seek any medical attention as his daily activities were not affected. One year before admission, the patient suffered from polydipsia (daily water intake around 5 L per day) and polyuria (urinary output around 6 L per day). Six months before admission, he experienced fatigue (unknown cause) and was anorexic. One month prior, the patient experienced cough and dyspnea. The cough and dyspnea were usually triggered by cold air. At this point, the patient was admitted to our hospital.

On admission, his vital signs were as follows: temperature, 36.0 °C; respiration rate, 18 breaths per minute; pulse, 73 beats per minute; and blood pressure, 120/60 mmHg. A painless, right submaxillary mass with a diameter of approximately 2 cm was palpable. He had reduced breathing sounds in both lungs without wheezing or rubs. No swelling was found in the lower limbs.

The main laboratory findings are shown in Table 1. Laboratory investigations showed elevated total serum IgE > 2500 IU/mL and peripheral eosinophil count of 0.62 × 10^9^/L, that was slightly increased. Considering dyspnea accompanied by elevated serum IgE and eosinophil levels, pulmonary function tests were performed. The findings of pulmonary function tests revealed obstructive ventilation function disturbance with a positive bronchial dilation test. A diagnosis of bronchial asthma was considered. To further investigate the potential cause of hypereosinophilia, bone marrow biopsy was performed. It indicated active granulocytic proliferation and an increased proportion of eosinophils.

**Table 1:** Main laboratory findings

Laboratory findings	Result	Reference value
*Complete blood counts*		
White blood cells (× 10^9^/L)	6.37	3.9–9.7
Neutrophils (× 10^9^/L)	3.4	1.9–7.2
Eosinophils (× 10^9^/L)	0.62	0.04–0.49
Red blood cells (× 10^12^/L)	3.0	4.3–5.8
Hemoglobin (g/L)	100	130–172
Platelets (× 10^9^/L)	292	135–350
*Urinalysis*		
Urine-specific gravity	1.003	1.003–1.030
Protein	Negative	Negative
Occult blood	Negative	Negative
*Biochemistry*		
Fasting blood glucose(mmol/L)	5.98	3.9–6.11
Urea nitrogen(mmol/L)	4.12	3–9.2
Creatinine (μmol/L)	59.2	59–104
Serum sodium (mmol/L)	140	136–145
Serum calcium (mmol/L)	1.17	1.15–1.29
Serum potassium (mmol/L)	3.95	3.5–5.5
*Immunology*		
IgE (IU/mL)	> 2500	1.31–165.3
IgG4 (g/L)	32.900	0.012–2.01
ANA	Negative	Negative (< 1:80)
ANCA	Negative	Negative
*Hormones*		
08:00 Cortisol (ug/dL)	15.62	6.2–19.4
08:00 ACTH (pg/mL)	36.93	7.2–63.3
FT4 (pmol/L)	11.29	9.01–19.05
TSH (uIU/mL)	2.0049	0.30–4.80
GH (ng/mL)	0.198	0.004–1.406
Prolactin (ng/mL)	12.82	2.64–13.13
Testosterone (ng/mL)	3.74	1.75–7.81
*Other relevant values*		
D-dimer (μg/L)	1109	0–252
TnI (μg/L)	< 0.01	0–0.04
BNP (pg/mL)	25.9	0–154.7
Plasma osmolality (mOsm/kg·H_2_O)	288	–
Urine osmolality (mOsm/kg·H_2_O)	140	–

**Fig. 2:**
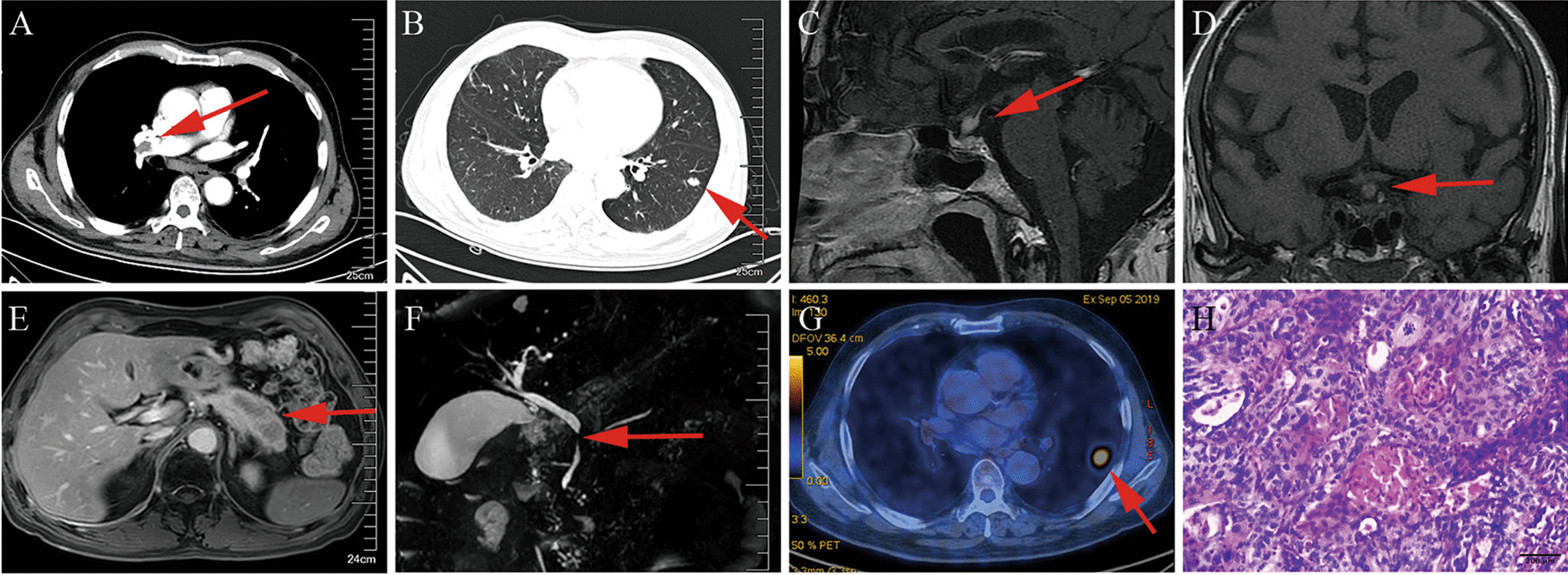
**A** Chest enhanced computed tomography showed filling defects in the right pulmonary artery (red arrow). **B** Chest enhanced computed tomography showed a nodule (1.4 cm × 0.9 cm) in the left inferior lobe basal segment (red arrow). **C, D** Enhanced MRI examination of the head showed pituitary stalk thickening in the sagittal (**C**) and coronal sections (**D**) (red arrow). **E** Pancreatic enhanced magnetic resonance imaging (MRI) showed swelling of the pancreatic tail (red arrow). **F** MR cholangiopancreatography showed local stricture of the pancreatic duct section of common bile duct (red arrow). **G** The positron emission tomography-computed tomography showed a nodule (2.3 cm × 1.5 cm) in the left inferior lobe with hypermetabolism (red arrow). **H** Histopathological examination of the left lung nodule: lung squamous carcinoma. Microscope type: ZEISS Microscope Model AXIO Lab A1, ZEISS camera Model Axiocam ICc 5 and ZEN software at a resolution of 144dpi and processed in adobe photoshop 21.0.2 at a resolution of 300 dpi. No downstream processing was utilized

*IgE* immunoglobulin E; *IgG* immunoglobulin G; *IgG4* Immunoglobulin G4; *ANA* anti-nuclear antibodies; *ANCA* Anti-neutrophil cytoplasmic antibodies; *ACTH* Adrenocorticotropic hormone; *FT4* Free thyroxine; *TSH* Thyroid-stimulating hormone; *GH* growth hormone; *TnI* Troponin; *BNP* Brain Natriuretic Peptide;

The level of serum D-dimer was elevated at 1109 μg/L. The patient had symptoms of dyspnea and elevated serum D-dimer level; therefore, we could not rule out pulmonary embolism. We performed chest enhanced computed tomography (CT), which showed filling defects in the right pulmonary artery and a nodule (1.4 cm × 0.9 cm) in the left inferior lobe basal segment (Fig. 2A, B). The brain natriuretic peptide and troponin I levels were within normal limits. There was no indication of right ventricular dysfunction on the echocardiogram. Additionally, the simplified Pulmonary Embolism Severity Index was scored as 0. Therefore, the patient was diagnosed with pulmonary embolism with low risk for early mortality. We suggested a needle biopsy for the left lung nodule for further evaluation; however, the patient refused due to concerns that withdrawal of anticoagulants would aggravate pulmonary embolism and that puncture might cause additional trauma.

Urinalysis showed reduced urine-specific gravity at 1.003. However, the renal function (serum BUN and serum creatinine), blood glucose, serum electrolytes (sodium, potassium, calcium), and serum hormonal (free T4, thyroid-stimulating hormone, 08:00 serum cortisol, 08:00 serum adrenocorticotropic hormone, prolactin and growth hormone, and testosterone) levels were within normal limits. Polydipsia, polyuria, and reduced urine-specific gravity were indicative of diabetes insipidus. Further investigations indicated decreased urinary osmotic pressure (140 mOsm/kg), which was lower than the plasma osmotic pressure (288 mOsm/kg). Enhanced MRI examination of the head showed pituitary stalk thickening (Fig. 2C, D). Polyuria improved following treatment with desmopressin acetate. Based on clinical manifestations, enhanced MRI of the head, and desmopressin acetate responsiveness, the patient was diagnosed with central diabetes insipidus.

The patient’s recurrent submaxillary mass, respiratory system involvement, central nervous system involvement, hypereosinophilia, and elevated serum IgE made us suspect systemic diseases. Further investigation showed elevated serum IgG4 levels (32.900 g/L). Antinuclear antibodies and anti-neutrophil cytoplasm antibodies were negative. Salivary gland emission computed tomography dynamic imaging showed impaired function in both submandibular glands. Pancreatic enhanced magnetic resonance imaging (MRI) and magnetic resonance cholangiopancreatography showed a swollen pancreatic tail and a local stricture of the pancreatic duct section of the common bile duct (Fig. 2E, F). We highly suspected that the patient might suffer from IgG4-RD. However, the patient refused to undergo any additional histopathological examinations. Therefore, we performed additional immunohistochemistry examinations of the eight-year-old specimens, which revealed that (1) IgG- and IgG4-positive cells were present, (2) The IgG4-positive cells per high-power field were around 20, and (3) The ratio of IgG4 + /IgG + was around 43% per high-power field (Fig. 1C, D). The pathology manifestations met the diagnostic criteria for IgG4-RD established in 2012 [11].

The patient was prescribed budesonide and formoterol (160/45 μg) twice daily for asthma. Rivaroxaban (15 mg twice daily for 3 weeks, followed by 20 mg per day for 3 months) was orally administered for pulmonary embolism. Desmopressin acetate tablets (Minirin®) were orally administered at a dose of 0.15 mg per day for central diabetes insipidus. We administered 30 and 50 mg per day of prednisone and iguratimod, respectively, for the IgG4-RD. The patient’s clinical condition improved significantly post-therapy, and the filling defects in the right pulmonary artery disappeared. Prednisone was gradually reduced. The serum levels of eosinophils, total IgE, and IgG4 decreased gradually.

**Fig. 3:**
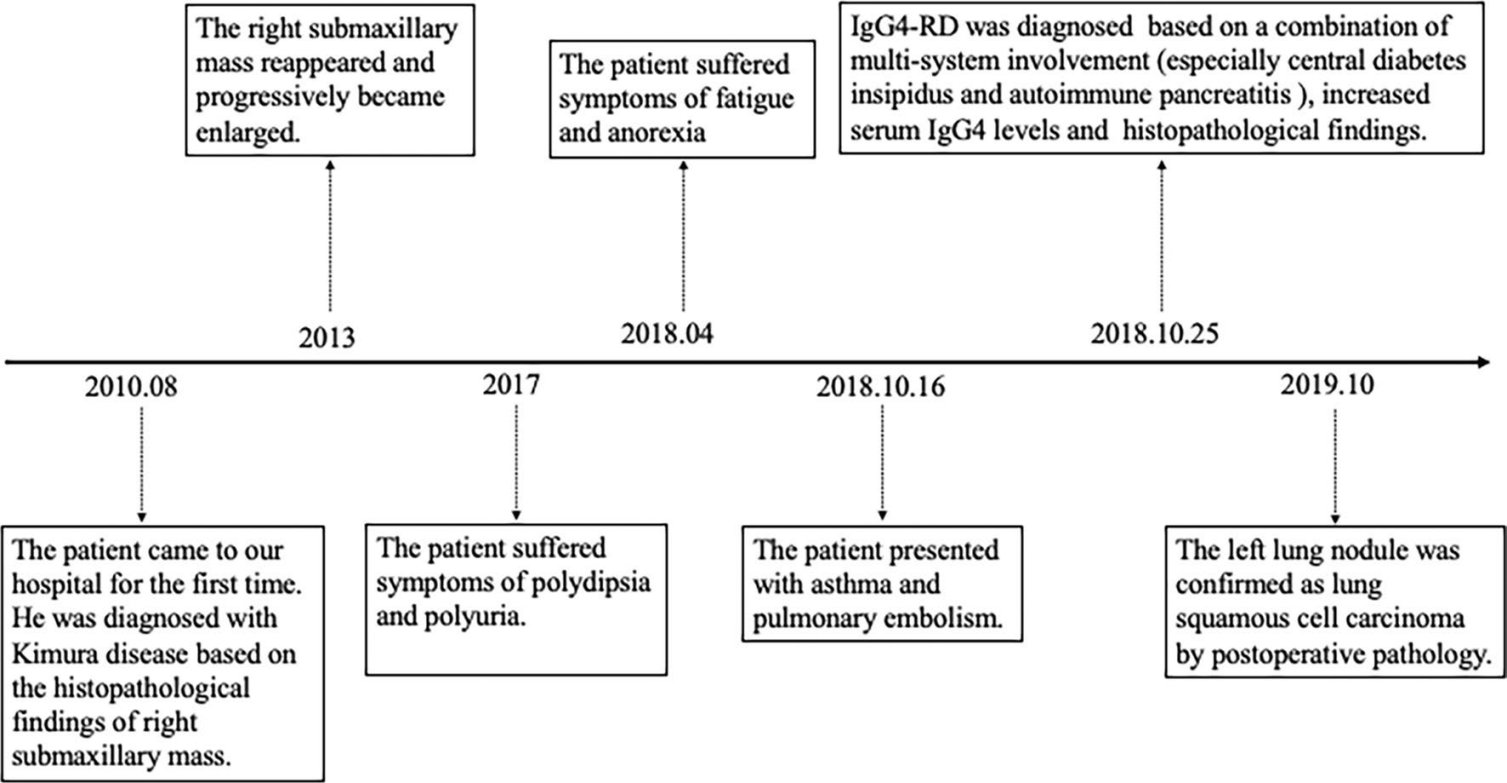
Timeline of the main clinical events

One year after his discharge from our hospital, the left pulmonary nodule was enlarged. The positron emission tomography-CT showed a nodule (2.3 cm × 1.5 cm) in the left inferior lobe with hypermetabolism (Fig. 2G). The patient underwent a wedge-shaped excision of the inferior lobe of the left lung. Pathological analysis revealed lung squamous carcinoma (Fig. 2H). He was diagnosed with lung cancer, T1cN0M0 stage IA3. Currently, the patient is in stable condition under treatment with glucocorticoids and immunosuppressants for IgG4-RD and remains free from recurrence of lung cancer in the follow-up chest CT scan. The timeline of the main clinical events is shown in Fig. 3.

## Discussion and conclusions

We report a case of concurrence of KD and IgG4-RD. The case possesses features of multiple system involvement accompanied by pulmonary embolism and lung carcinoma, which has not been reported before, suggesting that such cases are extremely rare.

Our patient showed clinical manifestations of both KD and IgG4-RD. The following clinical manifestations met the KD diagnostic criteria [3]: recurrent enlargement of submaxillary mass, elevated serum IgE levels, peripheral blood eosinophilia, and histopathological findings (lymphoid hyperplasia with germinal centers and eosinophil infiltration). The patient also met the criteria for IgG4-RD [11]: swelling of multiple organs (pancreas, hypophysis, and salivary gland), elevated serum IgG4 levels, and more than 10 IgG4-positive plasma cells per high-power field in immunohistochemistry.

KD and IgG4-RD can share certain common characteristics. Elevated serum IgE and hypereosinophilia are common in KD, but both have been reported in patients with IgG4-RD [12]. Although rare, asthma has been reported in patients with KD as well as IgG4-RD. Asthma in KD is related to IgE-mediated allergies [13]; 40–50% of patients with IgG4-RD have a history of allergic rhinitis and/or asthma [14], indicating a possible association between IgG4-RD and allergies.

Thromboembolism is a critical disease in clinical practice. The mechanism of pulmonary embolism in our patient is complicated; it can be explained considering the following: (1) Hypereosinophilia is strongly associated with thrombosis [15, 16]. We speculated that hypereosinophilia as a consequence of abnormal immune response was the main prothrombotic factor in this patient. (2) Malignant tumor formation is a high-risk factor for the development of deep venous thrombosis. When the patient was diagnosed with pulmonary embolism, he presented with a nodule in the left inferior lobe of the lung. One year after his discharge from our hospital, the nodule was confirmed to be lung squamous carcinoma. Therefore, we could not exclude the possibility of tumor involvement in the formation of pulmonary arterial thrombosis. (3) The pulmonary artery was involved in IgG4-RD, although this is rare. There is an earlier report of IgG4-RD case with pulmonary circulation involvement, presenting with pulmonary embolism [17].

Malignant transformation is not observed in KD. However, malignancies and IgG4-RD may be strongly associated. IgG4-RD increases the risk of cancer [10, 18, 19]. The pathogenesis may be related to the tumor immune escape possibly caused by the unique immunological effect of IgG4, which could destroy anti-tumor immunity [20]. Additionally, the chronic and persistent inflammation in IgG4-RD may promote tumor occurrence [10, 19]. Alternatively, IgG4-RD could be a para-neoplastic syndrome [21]. The expression of a particular antigen in tumor cells can cause an autoimmune reaction, which may induce the occurrence of autoimmune diseases [22]. Nevertheless, additional studies are needed to analyze whether malignancies promote the development of IgG4-RD. A series of abnormal immune reactions induced by unknown chronic allergen stimulation might be present in our patient, which may be the cause of the chronic and persistent inflammation involved in the occurrence of lung carcinoma. The definitive pathogenesis of IgG4-RD and malignancies in this case remains to be further studied. Our case indicates the importance of monitoring IgG4-RD patients for the development of malignancies during follow-up, especially in patients with obvious immune imbalance.

Understanding the potential common pathogenesis of IgG4-RD and KD may provide a certain basis for their co-occurrence. The pathogenesis of both IgG4-RD and KD may be associated with an unknown chronic allergen stimulation that can cause a series of abnormal immune reactions [1, 5]. Antigens induce T-helper 2 (Th2) cells [23]. The Th2-related cytokine, interleukin (IL)-10 is associated with the production of IgE and IgG, which can also induce the switch from IgE to IgG4 [12, 24]. In addition, Th2 cells produce IL-5, which is associated with eosinophilic activity [5]. These could explain the causes of concurrence of IgG4-RD and KD; however, further studies are still needed.

Reviewing the case, we find that the patient had a long course of disease, and the manifestations of the affected organs did not occur simultaneously. In addition, the patient first visited stomatology only with submaxillary mass and without any other clinical manifestations. This could have caused the specialist to neglect examination for systemic diseases, especially the immunohistochemical tests for IgG and IgG4 in the pathological tissue. Patients with KD are prone to relapse; the recurrence rate is 60 − 80% [3]. Confirmation of KD needs differential diagnosis with many other diseases, including angiolymphoid hyperplasia with eosinophilia, Hodgkin lymphoma, angioimmunoblastic T cell lymphoma, allergic granuloma, Langerhans cell histiocytosis, Castleman disease, and IgG4-RD [3]. Therefore, regular follow-up and careful differential diagnosis are important for patients with KD.

We report the various manifestations of a patient with concurrence of KD and IgG4-RD, an unusual combination of two rare diseases. This report highlights the importance of close monitoring for the development of malignancies in IgG4-RD.

## Data Availability

All data and material analyzed during this study are included in this published article.

## Abbreviations

IgG4: Immunoglobulin G4
IgG4-RD: IgG4-related disease
KD: Kimura disease
CT: Computed tomography
IgE: Immunoglobulin E
IgG: Immunoglobulin G
MRI: Magnetic resonance imaging
Th2: T-helper 2
IL: Interleukin

## References

[1] Kamisawa T, Zen Y, Pillai S, Stone JH (2015). IgG4-related disease. Lancet.

[2] Chen H, Thompson LD, Aguilera NS, Abbondanzo SL (2004). Kimura disease: a clinicopathologic study of 21 cases. Am J Surg Pathol.

[3] Zhang X, Jiao Y (2019). The clinicopathological characteristics of Kimura disease in Chinese patients. Clin Rheumatol.

[4] Kottler D, Barète S, Quéreux G, Ingen-Housz-Oro S, Fraitag S, Ortonne N (2015). Retrospective multicentric study of 25 Kimura disease patients: emphasis on therapeutics and shared features with cutaneous IGG4-related disease. Dermatology.

[5] Liu L, Chen Y, Fang Z, Kong J, Wu X, Zhang Z (2015). Kimura's disease or IgG4-related disease? A case-based review. Clin Rheumatol.

[6] Li J, Ge X, Ma J, Li M, Li J (2014). Kimura's disease of the lacrimal gland mimicking IgG4-related orbital disease. BMC Ophthalmol.

[7] Kotani H, Ohtsuka T, Okada S, Kusama M, Taniguchi T (2020). A case of IgG4-related disease presented with Kimura disease-like skin eruption, rheumatoid arthritis-like abnormality and interstitial pneumonia. Clin Exp Dermatol.

[8] Biedroń G, Kosałka J, Gąsior J, Milewski M, Gałązka K, Musiał J (2019). Kimura disease and immunoglobulin G4-related disease: a possible overlap in a patient with severe systemic symptoms. Pol Arch Intern Med..

[9] Lee JH, Kim JH, Lee SU, Kim SC (2018). Orbital mass with features of both kimura disease and immunoglobulin G4-related disease. Ophthalmic Plast Reconstr Surg.

[10] Tang H, Yang H, Zhang P, Wu D, Zhang S, Zhao J (2020). Malignancy and IgG4-related disease: the incidence, related factors and prognosis from a prospective cohort study in China. Sci Rep.

[11] Umehara H, Okazaki K, Masaki Y, Kawano M, Yamamoto M, Saeki T (2012). Comprehensive diagnostic criteria for IgG4-related disease (IgG4-RD), 2011. Mod Rheumatol.

[12] Wang X, Wan J, Zhao L, Da J, Cao B, Zhai Z (2019). IgG4-related disease with tracheobronchial miliary nodules and asthma: a case report and review of the literature. BMC Pulm Med.

[13] Dhingra H, Nagpal R, Baliyan A, Alva SR (2019). Kimura disease: case report and brief review of literature. Med Pharm Rep.

[14] Matsui S (2019). IgG4-related respiratory disease. Mod Rheumatol.

[15] Terrier B, Piette AM, Kerob D, Cordoliani F, Tancrède E, Hamidou L (2006). Superficial venous thrombophlebitis as the initial manifestation of hypereosinophilic syndrome: study of the first 3 cases. Arch Dermatol.

[16] Kanno H, Ouchi N, Sato M, Wada T, Sawai T (2005). Hypereosinophilia with systemic thrombophlebitis. Hum Pathol.

[17] Zhou Y, Shao L, Ruan W, Jin J, Xu H, Ying K (2019). Pulmonary vascular involvement of IgG4-related disease: case series with a PRISMA-compliant systemic review. Medicine (Baltimore).

[18] Kubo K, Yamamoto K (2016). IgG4-related disease. Int J Rheum Dis.

[19] Asano J, Watanabe T, Oguchi T, Kanai K, Maruyama M, Ito T (2015). Association between immunoglobulin G4-related disease and malignancy within 12 years after diagnosis: an analysis after longterm followup. J Rheumatol.

[20] Karagiannis P, Gilbert AE, Josephs DH, Ali N, Dodev T, Saul L (2013). IgG4 subclass antibodies impair antitumor immunity in melanoma. J Clin Invest.

[21] Shiokawa M, Kodama Y, Yoshimura K, Kawanami C, Mimura J, Yamashita Y (2013). Risk of cancer in patients with autoimmune pancreatitis. Am J Gastroenterol.

[22] Joseph CG, Darrah E, Shah AA, Skora AD, Casciola-Rosen LA, Wigley FM (2014). Association of the autoimmune disease scleroderma with an immunologic response to cancer. Science.

[23] McKelvie PA, Lyons B, Barnett G, Allen PW (2012). Kimura's disease in two Caucasians, one with multiple recurrences associated with prominent IgG4 production. Pathology.

[24] Jeannin P, Lecoanet S, Delneste Y, Gauchat JF, Bonnefoy JY (1998). IgE versus IgG4 production can be differentially regulated by IL-10. J Immunol.

